# Update on complications of synthetic suburethral slings

**DOI:** 10.1590/S1677-5538.IBJU.2016.0250

**Published:** 2017

**Authors:** Cristiano Mendes Gomes, Fabrício Leite Carvalho, Carlos Henrique Suzuki Bellucci, Thiago Souto Hemerly, Fábio Baracat, Jose de Bessa, Miguel Srougi, Homero Bruschini

**Affiliations:** 1 Divisão de Urologia, Faculdade de Medicina da Universidade de São Paulo, São Paulo, Brasil

**Keywords:** Urinary Incontinence, Polypropylenes, Postoperative Complications

## Abstract

Synthetic suburethral slings have become the most widely used technique for the surgical treatment of stress urinary incontinence. Despite its high success rates, significant complications have been reported including bleeding, urethral or bladder injury, urethral or bladder mesh erosion, intestinal perforation, vaginal extrusion of mesh, urinary tract infection, pain, urinary urgency and bladder outlet obstruction. Recent warnings from important regulatory agencies worldwide concerning safety issues of the use of mesh for urogynecological reconstruction have had a strong impact on patients as well as surgeons and manufacturers. In this paper, we reviewed the literature regarding surgical morbidity associated with synthetic suburethral slings.

## INTRODUCTION

**Figure 1:**
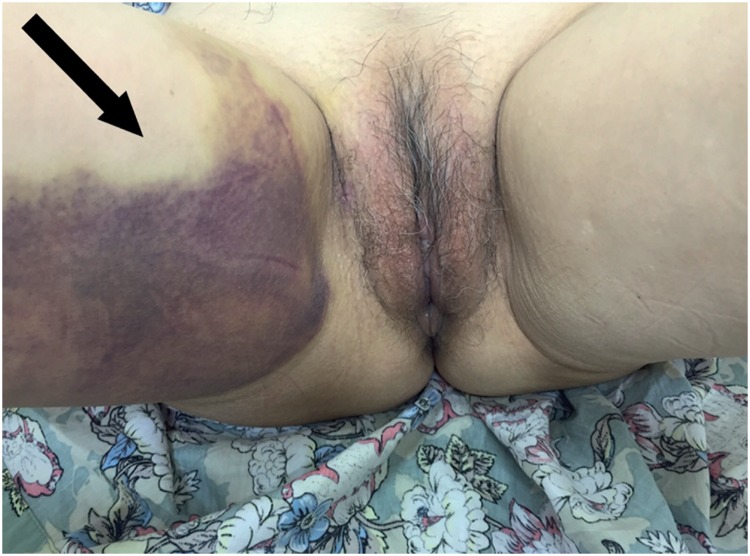
Haematoma of the right thigh (arrow) on post-operative day 3 of a transobturator SSS, with spontaneous resolution.

**Figure 2:**
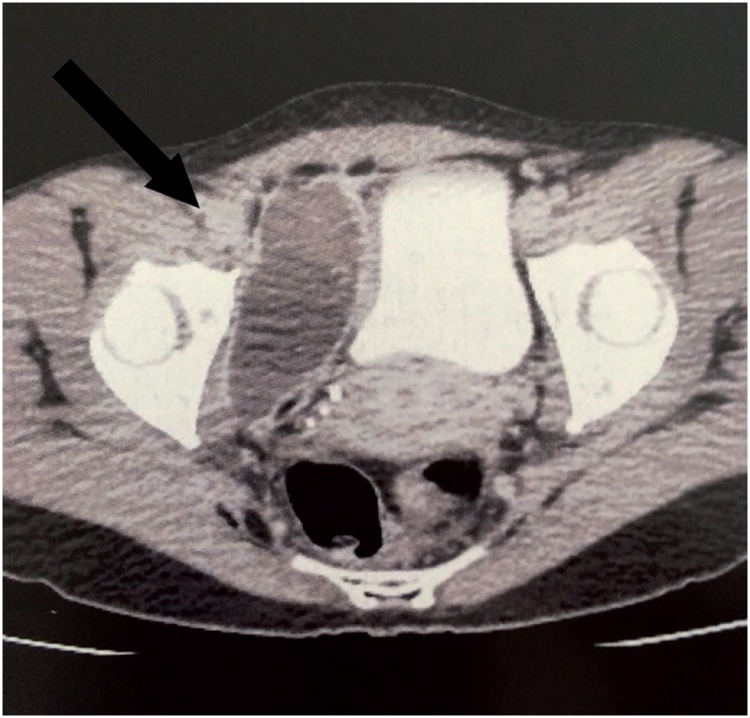
CT scan in the first postoperative day following a retropubic SSS demonstrates large pelvic hematoma (arrow) compressing the bladder laterally.

Stress urinary incontinence (SUI) is defined as the involuntary leakage of urine with effort or exertion, such as physical exercise, sneezing or coughing (1). Approximately 50% of all women experience SUI symptoms (1), and many of these women are sufficiently bothered by their symptoms to seek treatment from a physician. Pelvic floor muscle exercises and other nonsurgical treatments can be effective therapies, but many women choose to undergo surgery to treat their SUI symptoms. Suburethral synthetic sling (SSS) placement is the most common surgery currently performed for SUI and extensive data support their use for the treatment of female SUI. Compared to other surgical techniques, the advantages include shorter operative time/anesthetic need, reduced surgical pain and hospitalization time, and lower incidence of postoperative voiding dysfunction (2–10), The technique is based on the placement of a thin tape of synthetic mesh under the middle urethra which is passed through the retropubic space with a passing needle and exits the abdominal wall just above the pubis (Figure-1). It was introduced by Petros and Ulmsten in 1996 (11). By 2007, over 1.200.000 SSS had been performed worldwide and the numbers continue to increase exponentially (12, 13). A significant modification of the technique was the use of a transobturator route for the placement of the synthetic tape which was introduced by Delorme in 2001 (Figure-2) (14). The purpose of that was to eliminate the risks of complications associated with the passage of a needle in the retropubic space.

In the American Urological Association's opinion, any restriction of the use of SSS would be a disservice to women who choose surgical correction of SUI (15). However, despite the high success rates of the technique, a growing number of complications and adverse effects have been reported (9, 16–26). Recent reports indicate complication rates of 4.3% to 75% for the retropubic slings (2, 18) and 10.5% to 31.3% for the transobturator ones (Table-1) (27–30).

**Table 1:** Postoperative complication rates after syntethic suburethral sling surgery.

Complication	Retropubic	Transobturatory
**Bleeding**	0.7 to 8% ^(^ ^27^ ^,^ ^29^ ^,^ ^45^ ^,^ ^81^ ^,^ ^83^ ^)^	0-2% ^(^ ^27^ ^,^ ^29^ ^,^ ^36^ ^,^ ^37^ ^)^
**Bladder Injury**	0.7 to 24% ^(^ ^28^ ^,^ ^47^ ^)^	0-15% (12, 29, 48–50) ^(^ ^12^ ^,^ ^29^ ^,^ ^48^ ^–^ ^50^ ^)^
**Urethral Injury**	0.07 to 0.2% ^(^ ^44^ ^,^ ^45^ ^)^	0.1 to 2.5% ^(^ ^36^ ^,^ ^51^ ^)^
**Urethral Erosion**	0.03-0.8% ^(^ ^45^ ^,^ ^52^ ^)^	0.03 to 0.8% ^(^ ^45^ ^,^ ^52^ ^)^
**Intestinal Injury**	0.03 to 0.7% ^(^ ^18^ ^,^ ^63^ ^–^ ^65^ ^)^	0%
**Vaginal Erosion**	0-1.5% ^(^ ^28^ ^,^ ^29^ ^)^	0 to 10.9% ^(^ ^27^ ^,^ ^29^ ^,^ ^66^ ^,^ ^67^ ^)^
**UTI**	7.4 to 13% ^(^ ^4^ ^,^ ^27^ ^,^ ^28^ ^,^ ^37^ ^)^	7.4 to 13% ^(^ ^4^ ^,^ ^27^ ^,^ ^28^ ^,^ ^37^ ^)^
**Pain**	4% ^(^ ^75^ ^)^	9.4% ^(^ ^75^ ^)^
**Urgency “de novo”**	0,2% −25% ^(^ ^28^ ^,^ ^81^ ^,^ ^82^ ^)^	0 to 15.6% ^(^ ^27^ ^,^ ^28^ ^,^ ^83^ ^)^
**Bladder Outlet Obstruction**	6 to 18.3% ^(^ ^12^ ^,^ ^24^ ^,^ ^26^ ^,^ ^75^ ^,^ ^94^ ^)^	3.0-11% ^(^ ^12^ ^,^ ^24^ ^,^ ^26^ ^,^ ^75^ ^,^ ^94^ ^)^
**Urinary Retention**	4.1% −19.5% ^(^ ^2^ ^,^ ^28^ ^,^ ^29^ ^)^	2.7% −11% ^(^ ^28^ ^,^ ^29^ ^,^ ^37^ ^)^

Complications associated to SSS can be classified as immediate or late. Immediate complications include injuries during surgery as well as urinary retention and postoperative infections. Lesions may involve blood vessels, bladder, bowel, urethra, and nerves. Late complications occur weeks or months after surgery and include bladder outlet obstruction, urgency or urge-incontinence, recurrent urinary infections, erosion of the synthetic mesh to the urethra or bladder and extrusion of the tape to the vagina (31).

In a recent communication, the United States Food and Drug Administration (FDA) released an update on the safety and effectiveness of transvaginal placement of mesh (32). Although it was mainly directed to the placement of mesh for the treatment of pelvic organ prolapse, the use of mesh for the treatment of SUI was also included. The communication informed that mesh complications are not rare in transvaginal surgeries and may include serious adverse events (32). As a consequence, meshes from important manufacturers have been removed from the market. Moreover, women have been increasingly worried about the safety of SSS since they do not properly understand the differences between using mesh to treat pelvic organ prolapse as opposed to SUI.

## MATERIALS AND METHODS

There is a large body of evidence and review articles evaluating the complications of SSS. The goal of the current study was not to conduct a complete or systematic review or meta-analysis of the topic, but rather to perform a comprehensive overview based on published original and review articles augmented by a literature search. We performed a MEDLINE literature review using the “MeSH” (Medical Subject Heading) and “free text” protocols. The MeSH search was conducted with the following terms: “suburethral sling”, “surgical tape”, “urinary incontinence”, “female”. Multiple “free text” searches were performed using the following terms individually through all fields of the records: “sling”, “midurethral sling”, “transvaginal tape”, “transobturator tape”, “tension-free tape”. The search was restricted to the English language.

We divided the results in different topics regarding complications of synthetic suburethral slings, including bleeding, bladder and urethral injuries, bladder and urethral erosions, bowel injury, vaginal extrusion, urinary tract infection, postoperative pain, de novo urgency, urinary retention and bladder outlet obstruction.

## RESULTS

### Bleeding

The difficulty in reporting bleeding rates begins by defining this complication. It may vary from a simple hemorrhage during periurethral dissection that is self-limited and easily contained by compression to major vascular injuries with hemodynamic instability requiring aggressive treatment. The reports range from vaginal haematomas (Figure-1) to large vessel injuries with catastrophic outcomes (33). Insignificant haematomas may be common postoperatively after retropubic slings, and are occasionally found in 25% of patients undergoing magnetic resonance imaging (34). Haematomas of less than 100mL are rarely symptomatic, while the larger ones frequently cause abdominal discomfort (35).

Overall, bleeding rates vary from 0.7% to 8% for the retropubic slings and from 0% to 2% for the transobturator slings (24, 27, 36). Large series (28, 37–39) and metanalyses (24, 33, 40) have shown a significant lower risk of hemorrhagic complications with the transobturator technique. Deng et al. (41), reviewing twenty-eight series from 2001 to 2005, identified that 0.1% of the patients required blood transfusion.

Large haematomas in the retropubic space usually require surgical drainage (Figure-2), since aspiration appears to be ineffective (2, 35, 42). Injuries to major vessels during surgery require immediate surgical exploration with repair, ligation or reconstruction when possible (2). However, intraoperative bleeding is usually mild to moderate and under these circumstances transvaginal exploration is frequently ineffective and should be avoided. Since most of these cases are effectively managed by vaginal packing, the surgeon facing this complication should try and complete the procedure as fast as he can. Rarely, endovascular embolization has been used for the treatment of hemorrhagic complications of SSS surgery (43).

### Bladder and urethral injuries

**Figure 3:**
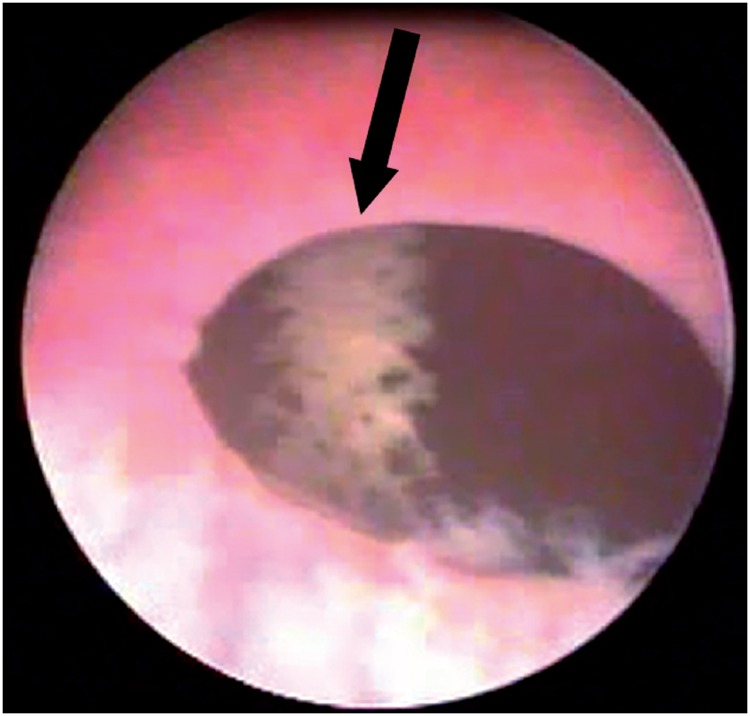
Cystoscopic view of sling mesh (arrow) in the bladder after a retropubic sling surgery.

Bladder injury during SSS surgery occurs in 2.7 to 6% of the patients (44, 45). Perforation by the needle is generally the cause of the lesion, which is thus more frequent at the lateral bladder walls (Figure-3). Rarely, the lesion may occur at the time of vaginal dissection and, in this circumstance, the bladder base is affected and the diagnosis is made by the observation of urine drainage at the injury site. Risk factors for bladder injury are previous anti-incontinence surgery, previous surgeries in the retropubic space and surgeon inexperience (46). For retropubic slings, the incidence ranges from 0.7 to 24% (28, 47), while for transobturator slings, the reported rates vary from 0% to 15% (12, 48–50). Most bladder perforations, if recognized during surgery, are treated by repositioning the needle and maintaining bladder drainage with a Foley catheter for 2-7 days (51).

Intraoperative urethral injury occurs in 0.07% to 0.2% for retropubic slings (44, 45) and 0.1% to 2.5% for transobturator (36, 52). It usually occurs during vaginal dissection of the paraurethral space and the diagnosis is made by visualization of the Foley catheter. However, inadvertent needle passage is also a possible cause. In these cases, the diagnosis is made during urethrocystoscopy. The lesion must be repaired immediately and placement of a synthetic sling at the same surgery is contraindicated (15). Prolonged bladder drainage with a Foley catheter (7-14 days) is recommended (51, 53, 54).

### Bladder and urethral erosion

Erosion is the extrusion of synthetic mesh to the lumen of the bladder or urethra, which occurs in the late postoperative period. Urethral erosion rates vary from 0.03% to 0.8% (45, 55). Typically, patients with bladder or urethral erosion present filling lower urinary tract symptoms such as urgency and urinary frequency, pelvic pain, dyspareunia, recurrent urinary tract infections, voiding symptoms and microscopic hematuria. Some cases with late diagnosis, may in fact be secondary to urethral or bladder injury during surgery that was overlooked. The eroded mesh may be calcified and present as a fixed bladder stone (Figure-4a). These patients may remain asymptomatic for several months, or present mild symptoms that increase gradually. The diagnosis of urethral or bladder erosion is confirmed by urethrocystoscopy (Figure-4b).

**Figure 4a:**
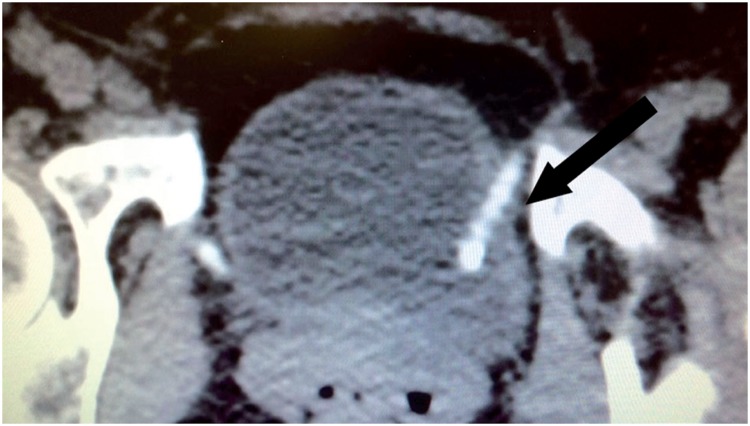
Pelvic CT scan shows calcified sling tape (arrow) eroding the bladder wall at the left side 2 years after a retropubic SSS.

**Figure 4b:**
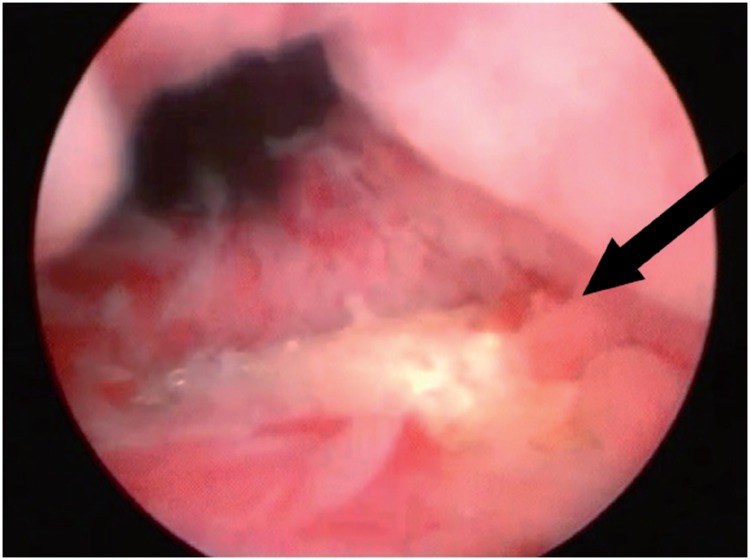
Mesh erosion in the urethra found in urethrocystoscopy two years after SSS (arrow).

**Figure 5:**
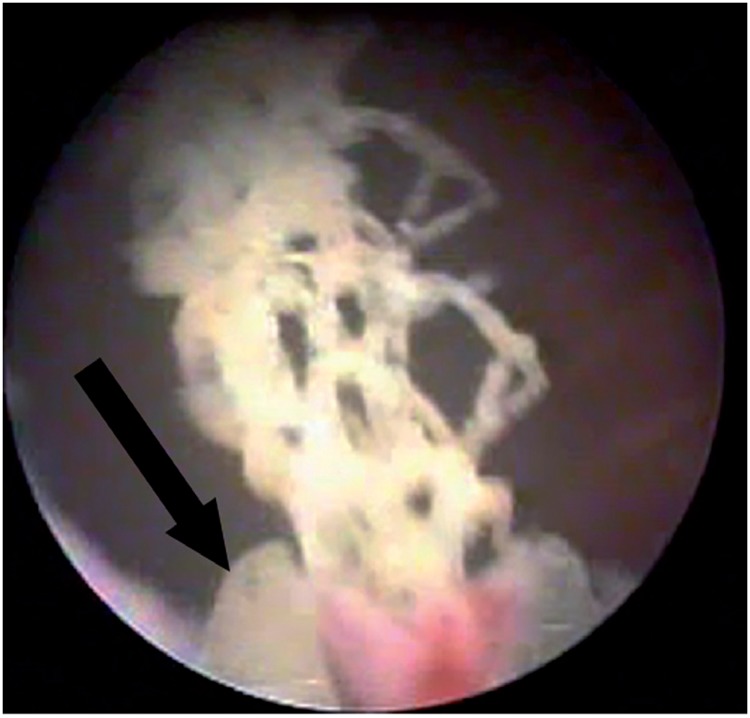
Endoscopic treatment of mesh erosion in the bladder using laparoscopic scissors (arrow).

Late urethral erosion is caused by excessive tension of the sling under the urethra, leading to progressive atrophy and subsequent erosion. Hypoestrogenism, prior vaginal or urethral surgery and pelvic radiation are conditions that determine worse urethral vitality and may contribute to a higher risk of erosion (55, 56). Treatment requires mesh removal and urethral repair (56). Total removal is usually performed by vaginal surgery. Laparoscopy may be used in selected cases of retropubic slings (57). Transvaginal partial removal is indicated for small erosions with little tissue loss and absence of infection. An endoscopic approach has also been proposed, consisting in removing the eroded mesh transurethrally and keeping a urethral catheter for 7-14 days (Figure-5) (58–60). In cases requiring urethral reconstruction with extensive tissue mobilization and long suture lines, a Martius flap should be associated to minimize the risk of a fistula (61, 62).

Voiding dysfunction after mesh removal is common. Starkman et al. evaluated 19 patients and reported that only 4 (21%) became completely asymptomatic after mesh removal. SUI recurred in 8 (42%) patients and only 9 (47%) considered themselves to be completely dry after surgery (63). Velemir et al. reviewed 17 cases of urethral erosion and obtained only 35.3% of complete urinary continence after transvaginal mesh removal (64). The same authors obtained a continence rate of 57.1% after endoscopic mesh removal. These results may reflect a milder severity of the erosion in patients who underwent endoscopic treatment. According to all authors, the simultaneous placement of a new SSS is contraindicated (15, 65). An autologous pubovaginal sling, however, may be considered (56, 63).

### Bowel injury

Bowel perforation is a life-threatening complication that has only been described with the retropubic technique. Few cases have been reported, with an estimated incidence of 0.03% to 0.7% (18, 66–68). The most important risk factor is previous pelvic surgery, which supposedly increases the risk of bowel fixation in the retropubic area. Clinical presentation may include abdominal pain, fever, malaise, leukocytosis, sepsis and bowel fluid discharge from the surgical wound.

Treatment consists of exploratory laparotomy for bowel repair and sling removal. A temporary bowel diversion may be warranted in cases with later diagnosis and bad tissue quality in which a primary repair is considered of high risk. It should be noted that the fact that the transobturator technique avoids the retropubic space makes this serious complication virtually impossible.

### Vaginal extrusion

Vaginal extrusion rates vary from 0% to 1.5% for the retropubic slings (28, 29) and from 0% to 10.9% for the transobturator (27, 69, 70). Regardless of the route used, the risk factors include inadequate closure of the vaginal incision, atrophic vaginal mucosa, local infection and unrecognized vaginal lesions during needle passage (17).

**Figure 6:**
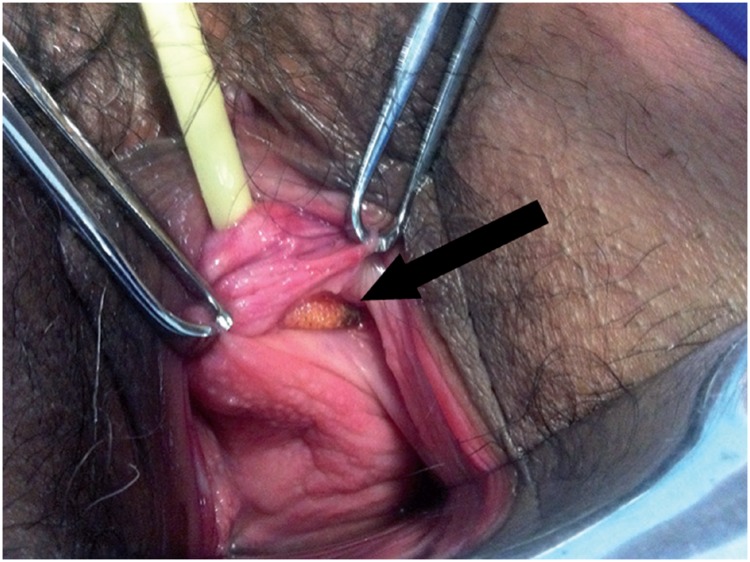
Vaginal extrusion (arrow) of mesh at the left anterolateral vaginal wall.

Vaginal extrusion (Figure-6) rates depend greatly on the type of synthetic mesh used. Polypropylene monofilament, malleable and macropore meshes are the standard meshes used in contemporary sling surgeries. They have been associated with lower extrusion rates in comparison to other meshes that were used in the past (71, 72). Those characteristics promote better tissue incorporation and facilitate local immune reaction reducing the risk of local infection. When extrusion is associated with infection, patients generally present with local pain, vaginal discharge and dyspareunia. The extruded mesh may be identified during vaginal examination. This presentation usually occurs within the first postoperative months and must be distinguished from that which occurs early and, in general, is caused by wound dehiscence, inadequate closure or inadvertent needle passage in the vaginal wall that remained unrecognized during surgery (66, 73, 74).

**Figure 7:**
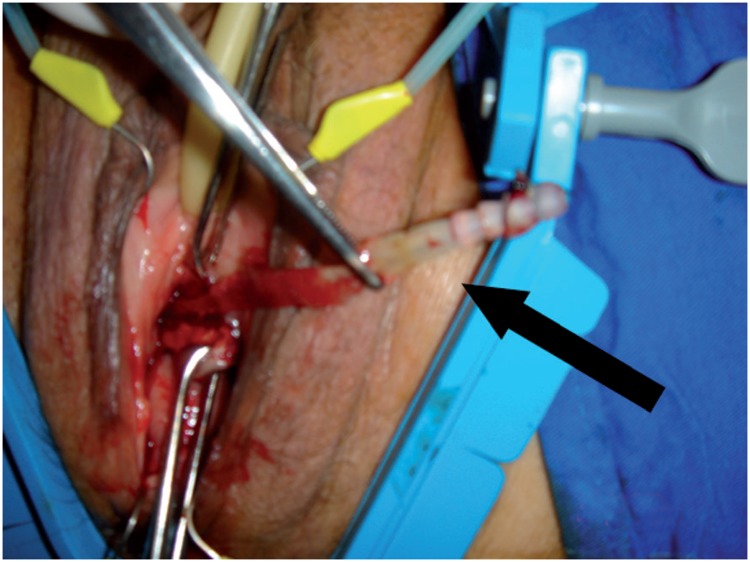
Transvaginal removal of an infected and extruded sling mesh (arrow).

If the extrusion is small and not associated with infection, conservative treatment and sexual abstinence may be adopted in order to permit second intention healing, with resolution in few weeks (75). Topical estrogen appears to improve the outcomes of conservative treatment (72). Authors recommend surgical removal of the eroded mesh segment if conservative treatment failed and when local infection is suspected (62, 76). This technique is accompanied by high resolution rates and the chance of recurrent stress urinary incontinence is very low. Reintervention for total mesh removal should be considered in cases of recurrence after the initial procedure (23). Erosions presenting with thorough purulent vaginal discharge, extensive vaginal inflammation or signs of systemic infection require aggressive treatment with total mesh removal (Figure-7). In this situation, the rates of recurrent stress urinary incontinence are approximately 20% (77).

### Urinary tract infection (UTI)

Recurrent UTIs after SSS surgery may represent a complication secondary to the presence of urethral/bladder erosion or bladder outlet obstruction. Anger et al. reported that 33.6% of the patients who underwent sling surgery had a UTI within the first 3 postoperative months, which increased to 46.7% within the first 12 months (66). Recently, other authors reported lower rates, ranging from 7.4% to 13%, with no significant difference between the retropubic and transobturator techniques (4, 24, 27, 28). Cases of recurrent UTI due to bladder outlet obstruction require surgical treatment, including mesh lysis or even urethrolysis, as needed. In cases of erosion, removal of the eroded sling and urethral reconstruction are indicated.

### Surgical Site Infection

**Figure 8:**
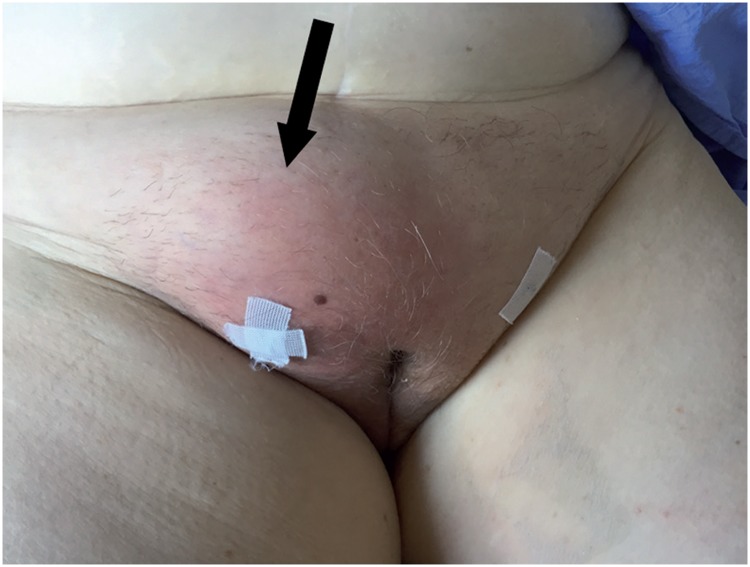
Large subcutaneous abscess (arrow) after transobturatory SSS treated with ultrasound guided puncture.

Surgical site infections after a SSS are rarely described and include superficial soft tissue infection and deep abscesso (78, 79). Clinical manifestations of these infections may include pain, tenderness, swelling (Figure-8) and fever, which begins during the first week after the procedure (80). It is easily identified during physical examination and the treatment is based on the use of large spectrum antibiotics (81). Occasionally, ultra-sonography, computer tomography or magnetic ressonance imaging may be used to evaluate the presence of an abscess and its exact extension as well as to guide its drainage (82). Although surgical site infections generally occur in the early postoperative period, cases of deep infections with delayed presentation have been described (82). Noteworthy, cases of severe necrotizing fasciitis after a SSS procedure has also been described (83, 84).

### Postoperative pain

Pain in the groin and thighs is one of the most common complications of suburethral sling procedures. The TOMUS trial, a prospective series of 597 patients followed-up for 12 months, comparing the transobturator and retropubic techniques, showed a lower incidence of so called neurological symptoms (pain) for the retropubic slings (4.0%) compared to the transobturator ones (9.4%) (85). In most cases, pain disappears within the first weeks after surgery, but it may persist for more than 4 weeks in 1% to 2.7% of the patients (12, 52, 70, 86).

Several factors may contribute to postoperative pain such as needle passage through pelvic muscles, infection, haematoma, and, more rarely, obturator nerve injury, a complication observed in less than 1% of the cases (24).

It was hypothesized that the inflammatory reaction of the sling material may lead to tissue retraction and hypertonia of the obturator muscle, which may simulate a pinched pudendal nerve, inducing groin and perineal pain (87, 88).

Treatment should be directed to the etiological factor and may vary from the use of common analgesics until drainage of an abscess or haematoma (24, 55, 89–91). In cases of severe or persistent infection, sling removal must be considered. Additionally, occasional patients that persist with pain despite adequate conservative treatment may also be considered for sling removal (23, 92).

### De novo urgency

Postoperative urgency is a common complication after SSS procedures, with rates ranging from 5.9 to 25% for the retropubic technique (93, 94) and from 0 to 15.6% for the transobturator slings (27, 95). When considering treatment for this condition, one should first exclude the possibility of sling erosion, local hematoma or bladder outlet obstruction. In these cases, treatment should be directed to the cause. If urethrolysis is required because of bladder obstruction, urgency symptoms may improve in up to 85% of the cases (96). When conditions such as sling erosion, urinary tract infection and bladder outlet obstruction have been ruled out, the principles of clinical management of urgency symptoms should follow those used for patients with the overactive bladder syndrome (97).

### Urinary retention

Urinary retention is a common early postoperative complication of all surgical procedures for SUI Its prevalence varies from 2.5 to 19.5% for retropubic (2, 55, 93, 98) and from 1.5 to 8.6% for transobturator slings (4, 24, 27). The majority of these patients present transient voiding dysfunction with spontaneous resolution in a period of 48 hours to 21 days. The initial management should be to provide a bladder emptying method (indwelling catheter or clean self intermittent catheterization). However, 0.3 to 4.5% of patients treated with a SSS persist with urinary retention for more than 4 weeks and require surgical mesh lysis (2, 4, 24, 27, 55, 93, 98).

### Bladder outlet obstruction (BOO)

Bladder outlet obstruction can be easily suspected when patients present with persistent urinary retention (longer than 4 weeks) or have overt symptoms of incomplete emptying, weak urinary stream and straining to void. However, a significant number of patients demonstrate less evident symptoms and the diagnosis often requires a high index of suspicion, frequently triggered by presentation with symptoms such as urgency, frequency and nocturia. The diagnosis of BOO in women may be challenging and should be made by taking into account the history, physical examination, imagining of the lower urinary tract and the urodynamic pressure-flow parameters (Figure-9a) (99, 100).

**Figure 9a:**
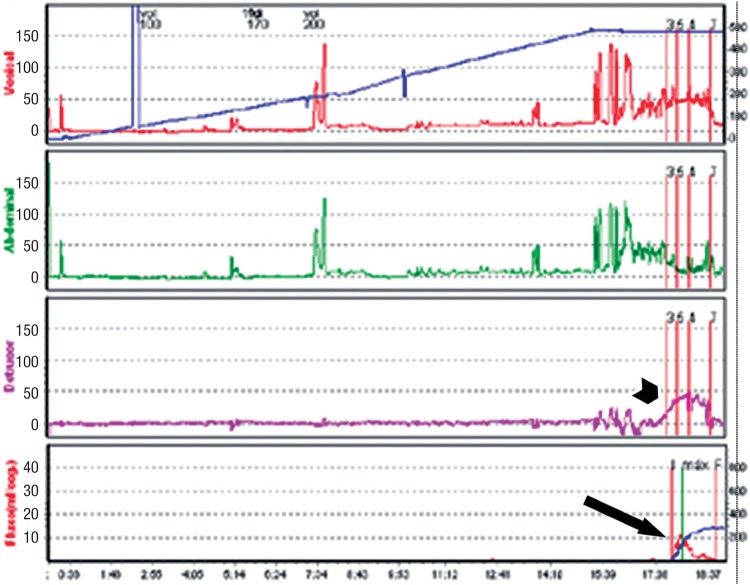
Urodynamics findings of a patient with BOO secondary to a retropubic SSS, showing high detrusor pressures (short arrow) and low maximum flow rate (long arrow).

In order to improve symptoms and to prevent progression of bladder dysfunction, postoperative BOO should be surgically relieved. The Tomus trial showed higher reoperation rates for treatment of voiding dysfunction in patients undergoing retropubic sling (2.7%) compared to those who underwent transobturator sling (0%; p=0.004) (85). The surgical options for BOO after a SSS surgery include sling incision (Figures 9b and 9c), sling lysis and partial removal and extensive vaginal or retropubic urethrolysis, with removal of the sling and disruption of the fibrosis surrounding the urethra and bladder neck (Figure-10). When outlet obstruction is diagnosed a long time after sling surgery, single mesh transection may be insufficient to improve BOO because of the possible fixation of the urethra to the pubis and the periurethral fibrotic process. In these cases, urethrolysis associated with mesh transection is recommended, with satisfactory results ranging from 70 to 85% and SUI recurrence in about 19% (96). If a second urethrolysis is needed, the resolution rate is about 92%, with recurrence of incontinence similar to the observed after the first one (22%) (101).

**Figure 9b:**
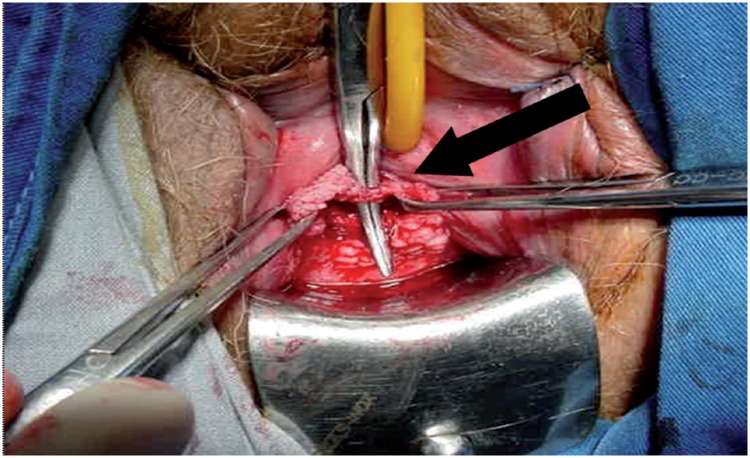
Sling incision (arrow) in the same patient after vaginal incision.

**Figure 9c:**
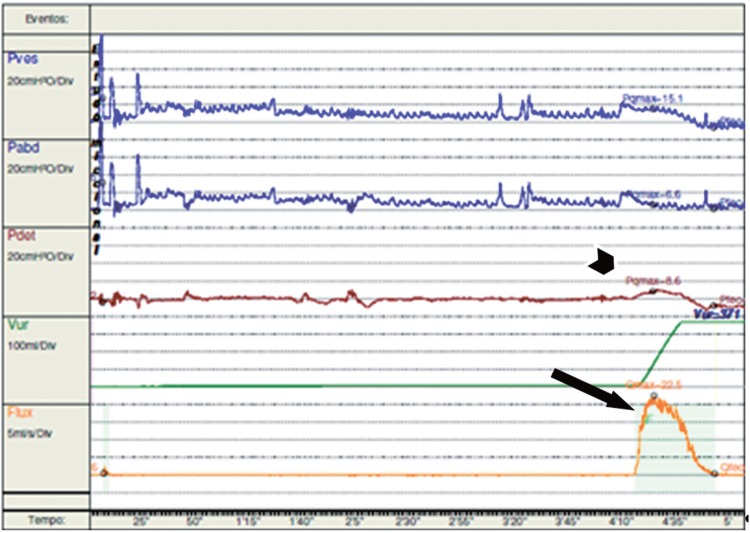
Postoperative urodynamics demonstrates resolution of the BOO, with low detrusor pressures (PdetQmax 8cm H20 - short arrow) and good flow (Qmax 42mL/s - long arrow).

### Medicolegal problems with vaginal mesh surgery

In addition to the medical problems, surgeons must be aware of potential litigation resulting from complications of vaginal surgeries with implantation of meshes. Since the FDA released a warning on the safety and effectiveness of trans-vaginal placement of meshes in 2011, the number of lawsuits has increased exponentially and has thus become a major concern to all vaginal surgeons. Given the potential risks involved, as well as the readily available legal recourse for patients who experience complications, it is important to deter litigation by appropriately counseling patients about the risks and documenting informed consent in the medical record (102–106).

## CONCLUSIONS

This review highlights the surgical morbidity of synthetic suburethral slings, which may include bothersome and even life-threatening complications. There is an increasing body of evidence to suggest that the number and severity of complications are underestimated, both by surgeons and patients.

As SSS surgery is the most common procedure performed for the treatment of female stress urinary incontinence, urologists and gynecologists must be aware of these complications, the strategies to avoid them and how to appropriately diagnose and manage the complications. Moreover, to lessen the chance of medicolegal problems, surgeons using transvaginal meshes should inform patients of potential complications associated with the products and document informed consent in their medical records.
